# Extract from an Address Read before the Indiana State Medical Society, by the President

**Published:** 1867-05

**Authors:** B. S. Woodworth

**Affiliations:** Fort Wayne


### Miscellaneous.

### Extract from an Address read before the Indiana State Medical Society, by the President,

DR. B. S. WOODWORTH, OF FORT WAYNE.

Let me describe a very few things which I consider charlatanic in the regular Physician, and the Physician above mediocrity too:

1st. There is the so-called author, who edits, as he calls it, a reprint of some English standard work—by writing a few unimportant notes and appending them to the book, and then having his name on the title page. Not to call any names, Philadelphia can boast of more physicians who have this cheap notoriety than any other city that I am acquainted with, though New York and some other cities contain some of the same sort. This I call gabbling to little purpose, or charlatanry; and certainly, although it may be rather harmless, it is not to be commended. There ought to be an international copyright law, to prevent these literary piracies. If it were not for being invidious or personal, I would like to name some of the worst specimens of this sort, but they will readily occur to you all.

Another charlatanic practice is puffing one's self, or having one's self puffed in the secular newspapers for the performance of some surgical operation, or for the performance of some wonderful cure. I very much fear that many of us have been tempted to connive at this practice, if not to do the very act. I recollect that I read not very long since, a long newspaper article of a really very good physician, that abounded in the most fulsome flattery and egotistical bombast, which described a wonderful cure performed by himself, after the patient had been given up by divers other physicians. We all know that the practice is reprehensible, and contrary to good manners and the code.

Another practice in our larger towns where the physicians prescribe, which ought to be condemned, is, the agreement between physicians and apothecaries that the physicians shall have a certain per cent. of all prescriptions which he may send to their shops, thereby tempting the physicians to write as many prescriptions of cheap, worthless drugs as he can without incurring suspicion of the collusion. I have known of many such cases, and I have myself often had such offers. No honorable physician should engage in any such dicker.

The practices of the real, downright quacks, I shall not undertake to describe. We are all too familiar with them. We know that their sole object is to live and get rich by imposing upon the public. They are all in the same stratum, whether in the form of a little pill peddler of infinitessimal sugar pretenses; "the mystical, scientific, radicalism of the drawing-room;" the rejuvenated Thompsonian fire eaters, now yclept eclectics; or "the common sense, scientific radicalism of the barn-yard;" that system which

Charles Lamb said was not new nor wonderful, but as old as the deluge, and like it, killed more than it cured, I. E. hydropathy; or the traveling mountebanks, abortionists, et al. They are all enemies of legitimate medicine, and ergo, of mankind.

And now the question is, how shall we combat them, or rather their systems and practices, for it will never do to have anything to do with them personally. That they can be combatted successfully, I verily believe; and that in the good time coming quacks of all sorts will be scarce, I doubt not.

Next to the education of the people universally in medical knowledge, a thing which will not probably be accomplished before the millenium, the most important means to put down quackery, as I before said, is to elevate the standard of our own education (and indeed I know of no other means to do it,) thereby pro ducing the results which I shall shortly mention. This proposi tion is a truism, as we all admit.

But the profession are not all agreed as to the best ways to arrive at this consummation so devoutly to be wished for. The American Medical Association has been discussing the subject ever since the formation of the society, without accomplishing a great deal, and our State society, has agitated the subject annual ly, without any very important results, though if we had the zeal of our learned friend, the late chairman of the Committee on Medical Education, who is certainly entitled to great credit for his perseverance under discouraging circumstances and difficulties, something more might perhaps have been accomplished.

But my chief design on this occasion is to mention what I conceive would be the effect of elevating the standard of medical education. One effect would be to lessen the number of physicians which, as everybody knows, is now, and for a long time has been superabundant, in fact, Legion. "Plato," says Burton, "made it a great sign of an intemperate and corrupt commonwealth when lawyers and physicians did abound;" and the Romans distasted them so much that they were often banished out of their city; as Pliny and Celsus relate, for 600 years not admitted. There is a far greater number than can get a decent living by the profession exclusively, and so they either drag out a miserable existence in poverty, or else unite with their drug-peddling (for it is nothing else) some other menial occupation. I know one who is a preacher (who will for this, probably, never be in any danger of having that "theological moxa" applied, with which Dr. Calvin killed our brother Servetus, as Holmes said,) "a justice of the peace, a tavern keeper, postmaster, hotel proprietor, attorney at law, physician, school teacher, &c., a Methodist minister, who can chew as much tobacco and drink as much whisky as any other man. And I am acquainted with several who have a nearly equal versatility of talent.

We all readily see that these things not only lower the profession in the estimation of the publis, but that they also totally unfit a man to practice our profession successfully; for no man can be a skillful and successful practioner of medicine who does not devote his undivided time and attention to this business, and no physician has any moral right to engage in any other business or occupation while he pretends to practice medicine.

Another effect of elevating the standard of Medical Education will be to make something more out of physicians than mere drug-prescribers; and by this I do not mean that they will ignore medicines altogether, by no means; but that they will use them in proper quantities, and at the proper times, and thus only. And here let me say, that whether we believe in all that the "Autocrat of the Breakfast table" has said, and float into his "currents or not," we can, I think, most of us heartily subscribe to one of his sayings in his late romance of Elsie Venner, where he says about the old doctor, "Some very silly people thought the old doctor did not believe in medicine because he gave less than certain poor, half-taught creatures in the smaller neighboring towns, who took advantage of the people's sickness to disgust and distate with all manner of ill-smelling, and ill-tasting, and ill-behaving drugs—to tell the truth, he hated to give anything noxious or loathsome to those who were uncomfortable enough already, unless he was very sure it would do good—in which case, he never played with drugs, but gave good, honest, efficient doses. Sometimes he lost a family of the most boorish sort, because they did not think they got their money's worth out of him, unless they had something more than a taste of everthing he carried in his saddle-bags." While I cannot subscribe to all the doctrines of Sir John Forbes, and while I think the mutural admiration Professors of the American Athens to be somewhat ultra, I yet think that they have done incalculable good to legitimate medicine, by unmasking that absurd delusion, Homeopathy, as well as other quackeries. And if I mistake not, already a majority of the most enlightened physicians are with Professor Hughes Bennett, as opposed to the more heroic party.

